# Is the delivery of a quality improvement education programme in obstetrics and gynaecology for final year medical students feasible and still effective in a shortened time frame?

**DOI:** 10.1186/s12909-017-0927-y

**Published:** 2017-05-26

**Authors:** Bridget Kool, Michelle R. Wise, Roshini Peiris-John, Lynn Sadler, Faith Mahony, Susan Wells

**Affiliations:** 1 Section of Epidemiology and Biostatistics, School of Population Health, University of Auckland, Auckland, New Zealand; 2 Department of Obstetrics and Gynaecology, University of Auckland, Auckland, New Zealand

**Keywords:** Quality improvement, Undergraduates, Medical, Clinical audit, Medical education

## Abstract

**Background:**

Teaching clinical audit skills to nascent health professionals is one strategy to improve frontline care. The undergraduate medical curriculum at the University of Auckland provides improvement science theory and skills in Year 5 teaching, and the opportunity to put this into practice during an Obstetrics and Gynaecology (O&G) clinical attachment in Year 6. In 2015, a revised medical school curriculum at the university resulted in a planned reduction of the O&G attachment from five weeks to four, necessitating revision of the Year 6 Quality Improvement (QI) project. The aim of this study was to evaluate if the revised programme provided an important experiential learning opportunity for medical students without imposing an unsustainable burden on clinical services.

**Methods:**

Based on a CIPP (Context/Input/Process/Product) evaluation model, the study was conducted in several stages to get a sense of the context as the new programme was being planned (Context evaluation), the feasibility of an alternative approach to meet the educational need (Input evaluation), the implementation of the revised programme (Process evaluation) and finally, the programme outcomes (Product evaluation). We used multiple data sources (supervisors, students, academic administrators, and hospital staff) and data collection methods (questionnaires, focus groups, individual interviews, consultative workshops, student reports and oral presentations).

**Results:**

The context evaluation revealed the Year 6 QI programme to be valuable and contributed to O&G service improvements, however, the following concerns were identified: time to complete the project, timely topic selection and access to data, recognition of student achievement, and staff workload. The evaluation of the revised QI project indicated improvement in student perceptions of their QI knowledge and skills, and most areas previously identified as challenging, despite the concurrent reduction in the duration of the O&G attachment.

**Conclusions:**

Applying the CIPP model for evaluation to our revised QI programme enabled streamlining of procedures to achieve greater efficiency without compromising the quality of the learning experience, or increasing pressure on staff. A four week clinical rotation is adequate for medical educators to consider opportunities for including QI projects as part of student experiential learning.

**Electronic supplementary material:**

The online version of this article (doi:10.1186/s12909-017-0927-y) contains supplementary material, which is available to authorized users.

## Background

Rising healthcare costs, variations in quality and practice, lack of accountability and inequalities in health have led to an increased interest globally on health care quality and patient safety. In order that the medical curriculum reflects contemporary trends in medicine and remains relevant, undergraduate and graduate programmes worldwide have developed clinical teaching of quality improvement (QI) [1, 2]. Most of these programmes are designed to engage physician trainees to improve the care of the patients and the system in which they practice. Professional colleges have also taken up the challenge and now require their fellows to undertake QI projects (QIPs) for their ongoing professional development and vocational accreditation. Whilst providing training in effective QI skills has typically received little attention in medical curricula, the few published reports have demonstrated improved knowledge, processes of care and patient outcomes with the curricula generally being well accepted [1–6].

Teaching QI to clinicians, improves collaborative skills and provides the opportunity to work with colleagues from other disciplines [1]. The engagement of both educational and organisational stakeholders is identified as critical to the success of curricula in medical training in QI and patient safety [2]. Integral to this is the development of sufficient expert faculty capacity to ensure clinical supervisors have the skills and capacity to support QI training during clinical placements. Clinicians who have a foundational knowledge in QI function are imperative as train-the-trainers [3, 4].

Within postgraduate teaching programmes, time and clinical commitments, lack of engagement by clinical teams in QI activities, availability of suitable faculty for mentoring, and an organisational culture reluctant to change are identified as barriers in the development of postgraduate medical trainees into future leaders for QI and patient safety [7]. It is hoped that incorporating QI into undergraduate medical curriculum may help break some of the barriers, as a critical mass of clinicians with an appreciation of, and skills in, QI emerges.

Medical students are expected to increasingly rely on experiential learning as they progress through to clerkships and transition to being qualified doctors [8]. Experiential learning or learning to practice from experience gained within real life (workplace learning) builds on situativity theory where knowledge, thinking (cognition), and learning are situated in experience [9]. A situated approach improves a student’s ability to apply knowledge gained in one context to problems encountered in another. While traditionally experiential learning has been positioned primarily in the context of one-on-one patient care, the application of experiential learning theories in learning QI practices within the undergraduate medical curricula is relatively new. The importance of experiential learning in QI curricula and the need for documenting examples of QI learning that is integrated into day-to-day clinical work has been previously emphasised [10].

Medical education programmes are characterised as complex systems, affected by factors both internal and external to the programme [11]. The success of a QI education programme situated within multiple clinical training sites will inevitably be influenced by student, trainer, hospital and university stakeholder characteristics, their relationship to each other, and the environment in which they act.

Complexity theory recognises the importance of programme context and allows the accommodation of the numerous uncertainties within educational programmes [12]. Informed by complexity theory and embracing the complexity of educational processes, the CIPP (Context/Input/Process/Product) evaluation model [13] allows the examination of change in a medical education programme, addressing all phases: planning, implementation, and a final retrospective assessment. The model helps educators focus on improvement by considering the processes involved and multiple data collection methods based on evaluation questions being addressed.

The undergraduate curriculum at the School of Medicine of the University of Auckland provides improvement science theory and skills (such as QI frameworks and measurement strategies for service improvement) in Year 5 teaching and the opportunity to put this into practice during an Obstetrics and Gynaecology (O&G) clinical attachment in Year 6 by performing a QIP based on the Royal Australian and New Zealand College of Obstetrics and Gynaecology (RANZCOG) quality cycle [14]. Students work alone or in pairs during their O&G clinical attachment at one of eight clinical training sites (public hospitals) in New Zealand. Based on clinical recommendations, a literature review and evidence-based guidelines, students select a best practice standard/s for care for their chosen topic, measure service performance, seek to understand the problems contributing to the evidence-practice gap, and propose suggestions for change. Assessment includes a written report and an oral presentation. Over 1000 projects have been completed since the training’s inception.

A recent review of the medical school curriculum at the University of Auckland resulted in a planned reduction of the O&G attachment from five weeks to four (commencing in 2015), and necessitated a revision of the Year 6 QIP in the midst of clinical training demands. The aim of this study was to evaluate the feasibility of delivering a revised Year 6 QIP programme in a shortened time frame. We were interested in determining: 1) if there was a place for a QIP in a brief Year 6 O&G clinical attachment, and if so, why?; and 2) if shortening the O&G attachment would impact on students’ ability to execute the QIP and/or impose an unsustainable burden on clinical services? Adopting a CIPP evaluation model enabled us to get a sense of the context as the new programme was being planned, the feasibility of an alternative approach to meet the educational need and how best to bring about any required changes (Input evaluation), the implementation of the revised programme (Process evaluation) and finally, the programme outcomes (Product evaluation).

## Methods

The study involved all O&G QIP supervisors, Year 6 medical students attached to O&G during the study period, academic administrators, and hospital staff (data managers and QI staff) at all eight clinical training sites. The evaluation questions based on the CIPP model that guided the approach to our evaluation are listed in Table 1.

**Table 1:** Framework for evaluation of the revised Year 6 quality improvement project

CIPP components	Evaluation questions	Data collection method
Context	What is the educational need? What are the impediments to meeting needs? What expertise, services, or other assets are available? What relevant opportunities exist?	Student focus groups and individual interviews Survey questionnaire
Input	What are the potential approaches to meeting the identified educational need? How feasible is each of the identified approaches, given the specific educational context of the need?	Consultative workshop Telephone interviews
Process	How was the programme implemented? What did participants think about the quality of the process?	Student focus groups Survey questionnaire QI presentations and reports
Product	What are the positive and negative outcomes of the programme? What are the implications of programme outcomes? How effective was the programme?	

(Adapted from Frye and Hemmer, 2012) [12]

### Data collection

#### Context evaluation

The needs, problems, assets, and opportunities relevant to the QIP programme were assessed though a survey questionnaire, semi-structured focus groups and interviews, and evaluation of the quality of student reports and presentations.

Four semi-structured focus group discussions were conducted with Year 6 medical students (two in each of the main centres), to gain more insight into the domains being explored in the student questionnaire. Two individual interviews took place in two of the smaller centres where there were insufficient students placed for a focus group to be conducted. The interview schedule included questions relating to the QIP learning outcomes, content, value and transferability, workload and time management, strengths and improvements. In addition, the focus groups and interviews explored whether students perceived their expectations regarding the QIP had been met. A total of 32 out of 34 students participated in the focus groups and interviews (Table 2).

**Table 2:** Participant numbers in the evaluation of the revised Year 6 quality improvement (QI) project

	Total	Number who completed the questionnaire	Number who participated in the focus groups/interviews
Cycles 2 and 3 from 8 sites in 2013			
Students	34	21	32
Clinical supervisors	63	31	Not applicable
Hospital staff (data managers and QI staff)	16	13	Not applicable
Academic administrators	3	3	Not applicable
Cycles 6 and 7 from 6 sites in 2013			
Students	33	11	30
Clinical supervisors	12	8	Not applicable
Hospital staff (data managers and QI staff)	Not applicable	Not surveyed	Not applicable
Academic administrators	Not applicable	Not surveyed	Not applicable

The domains explored in and findings from the focus group sessions and interviews and a review of relevant literature [15, 16] informed the development of stakeholder specific questionnaires. The domains of interest were the same as those used for the focus group discussions. Some open ended questions particularly in relation to workload and time management, strengths and improvements were also included in the questionnaire. All O&G QIP supervisors, Year 6 medical students attached to O&G during the study period, academic administrators, and hospital staff (data managers and QI staff) were invited by email to complete their specific questionnaire (via SurveyMonkey) on completion of cycles 2 and 3 of the O&G attachments in 2013. Participants were given one week to complete the survey, with two email reminders sent. Students (*n* = 21/34), clinicians (*n* = 31/63), hospital staff (*n* = 13/16), and academic administrators (*n* = 3/3) representing the eight clinical training sites completed the questionnaire.

#### Input evaluation

To maintain maximum responsiveness to the shortened QIP programme needs, a consultative workshop was held to identify how best to bring about the needed changes. All O&G clinicians who supervised QIPs, University of Auckland Year 6 medical students in O&G attachments (cycles 2 and 3 in 2013), academic administrators, and hospital staff (data managers and QI staff) were invited to participate in a consultative multidisciplinary workshop held in May 2013. The aim of the workshop was to obtain feedback on the feasibility of the proposed four-week time frame, understand hospital-specific issues relevant to the QIP, and generate ideas for improving the programme without affecting the quality of the learning experience or increase pressure on clinical supervisors and other hospital staff.

The workshop feedback was recorded by the participants on flip charts and handed over to the researchers at the end of each session. The information collected was then used to describe issues relevant to the QIP and how the programme could be improved.

Nineteen people attended the workshop, an additional four who could not attend were interviewed via telephone. Field notes were taken during the telephone interviews and incorporated into the workshop findings. The 23 participants represented all eight training clinical settings.

Given the variation of implementation of the project by hospital site, the research team worked closely with students, clinicians and hospital QI staff to develop a more consistent programme, address concerns raised and ensure feasible completion in the shortened four-week time frame. A revised QIP model was developed drawing on the findings from the context evaluation, outcomes of the workshop and evidence from published literature, and trialled in the last two cycles (cycles 6 and 7) of 2013. Students’ usual O&G clinical placement of five weeks was reduced to four weeks which meant they had only four weeks to complete their QIP. In the redundant fifth week, students completed any outstanding required clinical skills for the placement.

#### Process and product evaluation

The process and product evaluation of the revised QIP programme was conducted simultaneously at the end of each of the last two cycles (cycles 6 and 7) of the O&G attachments, in late 2013 by means of by means of student focus groups and the same baseline online surveys for students and clinical supervisors. The questions for student focus group sessions pertained to both process and outcome; hence the findings are presented together. Individual interviews were not required in this phase as no students had been placed in the two smaller centres. The research team, in discussion with the hospital staff (data managers and QI staff) and academic administrators, elected to not resurvey these two groups as it was felt the revisions made to the QIP had not altered their roles and responsibilities and therefore unlikely to alter their questionnaire responses. Out of the 33 eligible students, 11 participated in the survey questionnaire, and 30 in the focus groups. Eight out of 12 clinical supervisors responded to the survey.

The programme’s implementation was also assessed through other documented programmatic processes. The degree to which the QIPs had met the targeted educational needs was assessed by a critique of the quality of student oral presentations of the QIP (including feasibility of the project and audit methodology) to clinical and academic assessors, and the QIP written reports (*n* = 33) (cycles 6 and 7, 2013). Assessors evaluated the presentations and reports were using a pre-agreed template with several criteria related to: clear articulation and justification of the QI question, aims of the audit, definition of standard, description of methods, results, interpretation of findings, limitations and recommendations. The results of this assessment were compared with the quality of student presentations and written reports (*n* = 34) assessed at the time of context evaluation (cycles 2 and 3, 2013).

The process and product evaluations were conducted through the same student focus group sessions and survey questionnaires. As the focus groups and interviews had questions pertaining to both process and outcome, the findings are presented together. The online questionnaires used at baseline were administered to Year 6 medical students and the clinical supervisors on completion of cycles 6 and 7 of the O&G clinical attachments for 2013. As was the case in the context evaluation phase of the project, participants were given one week to complete the survey with two email reminders sent. Focus groups were also conducted with students at the end of their clinical attachment.

### Data analysis

Descriptive statistics were used to summarise the quantitative data from the questionnaires. Focus groups and interviews were recorded and transcribed. The transcribed data and free text survey were studied and relevant quotes coded and analysed using the general inductive approach [17] that enabled raw data to be condensed into a brief summary format and to establish links between the research objectives and the summary findings. The inductive coding process involved the close reading of the text and the consideration of its multiple meanings and then categorising these texts. The general categories were derived from the evaluation objectives and within each category, subtopics, including new insights and contradictory points were identified. Quotations that convey the essence of the categories were then extracted. Coding consistency checks were carried out by members of the research team and some clinical supervisors commenting on research findings, interpretations, and conclusions.

In the Results section below, identities are protected through the use of anonymised, unique identifiers for individual participants and hospital sites. These appear in parentheses after each direct quotation.

## Results

### Context evaluation

The categories identified in the student focus group and interview responses in the context evaluation were educational need and impediments to meeting the needs (as indicated in the quote below), and expertise and opportunities.

*Appreciation of what it means to undertake an audit - it can be a marathon (Student W1).*

Exposure to people working in medical records was noted by one student as a new and useful experience.

The questionnaire indicated that the majority of students (91%), clinicians (85%) and hospital quality improvement and data management staff (70%) agreed the project provided students with useful insights into QI in O&G. Similarly, 81% of the students, 86% of the clinicians and 82% of hospital QI and data management staff agreed that the skills learnt would be important for their future.

*.. students get really into their topic and ask midwives and nurses what actually happens on the floor, they get a completely different perspective on patient care (Clinician A8).*

Most clinicians (*n* = 23) and half of the students agreed the QIP was a necessary component of the curriculum. However, both parties commented on the potentially competing interests of ensuring students’ QIPs were completed as well as obtaining sign off on the clinical skills that are required to be demonstrated during students’ O&G clinical placement.

Three-quarters of clinicians agreed that involvement with the QIP was satisfying and that the QIP provided value to the O&G service and the organisation.

*Young enthusiastic minds bring fresh ideas and potential solutions to problems, resulting in better patient care. (Clinician W12).*

Whilst only one student referred to inadequate support and guidance to achieve the learning objectives, half of the clinicians believed they were able to give adequate support to students. One third of clinicians and over half of hospital staff encountered challenges with regards to workload and time commitment. In addition, hospital staff reported that assisting students to access data placed a burden on their resources.

The majority of students had their topic suggested by a clinician.

*We have to suggest topics to them. They do not know enough about what is important and what is feasible when they start the attachment. (Clinician WH5).*

Nearly half the students reported that time constraints affected their QIP learning experience and limited their ability to complete it to a sufficient standard. While it was anticipated the project would be completed during the weekly academic half-day, the average time spent was 7.0 h per week.

On average, clinicians spent 2.3 h per attachment assisting students with their QIPs. The majority of students, and just over half of clinicians and hospital staff, indicated they had encountered issues with accessing relevant data/records for the QIPs.

### Input evaluation

Implementation of the QIP varied considerably across the hospitals. While some variation was due to local context, there were differences in the consistency of teaching and support. A mid-cycle debrief was seen to be a useful activity to ensure students were on track, yet occurred only at a few hospitals. Some clinicians and hospital staff felt the time they put into supporting student QIPs was not valued by their organisation. Conversely, others stated the O&G service improvements resulting from the QIPs were highly valued, as was their time spent with students.

Teaching materials identified that could support students included; online “how-to” documents; an annually updated Excel spreadsheet of completed projects; prior student reports; and an introductory video for both students and clinicians describing the project and audit process. Students considered these to be valuable resources, and suggested adding ‘tips and hints’ from previous students.

Deciding the audit topic as soon as possible was felt to be a critical factor and the list of previous QIP reports was considered useful for topic ideas and methodology. While some clinicians felt that supervisors were more likely to engage with the students if the audit topic was considered useful to the O&G service, others maintained that the project’s primary objective was for students to learn QI skills.

In some hospitals, students asked other clinicians for assistance rather than their designated supervisor. This practice exposed a lack of knowledge of audit methodology among other hospital doctors. Issues were raised about the time and cost of obtaining paper-based patient records in one hospital. In some hospitals with electronic records, students lacked access authority. An urgent issue raised by hospital staff was storage and confidentiality of data.

The use of student QIP reports by clinicians varied from not-at-all to being fully integrated into service improvement activities such as being forwarded to service clinical leads, and informing clinical governance committees. Some hospitals stored prior reports on an intranet to increase accessibility.

Despite the issues raised, it was felt that the project was valuable and that with some modifications it would be feasible to complete the QIP in four weeks instead of five.

### Process and product evaluation

The following key changes that were implemented based on the findings from the context evaluation and the consultative workshop were documented:

- Students were encouraged to work in groups of two or three, to focus on one standard of care, and to use feasible “rule of thumb” sample sizes.
- The QIP introductory video was provided to all supervisors.
- Supervisors were encouraged to profile written reports for their hospital.

In addition, the following resources were developed and implemented.

1. Topic Selection Form: completed by the student and submitted to their supervisor/s by the end of the first week and same-day feedback given (Additional file 1). The aim was to ensure students were on track early on and to guide the scope and focus of their QIP topic.
2. Written Report Template: standardised the way students presented their report with all relevant information included (Additional file 2). The aim was to guide students on expectations and provide the information to hospital services and clinical governance groups in a useful and concise format.
3. ‘Tips and Hints’ page: an on-line resource including a student discussion board containing generic and site-specific tips.

Quality of the process and the effectiveness and implications of the programme were overarching categories that emerged from the student focus groups in the process and product evaluation stages.

*It was good … it’s not like you did half your audit and then you realised that there was a big problem and you had to go back and do it again. (Student WK7)*

**Fig. 1:**
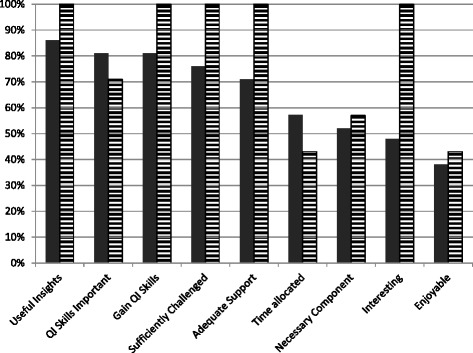
Student perceptions on QIPs before and after revision of the Year 6 QI programme

The survey indicated that students were on average more positive about the revised QIP programme than at the point of context evaluation (Fig. 1) with over half of students (55%) agreeing that four weeks was sufficient to achieve the desired learning objectives. The average number of hours spent on project per week had however increased to 7.4 (cf. 7.0 at baseline).

Fewer students reported problems accessing patient records (27% cf. 42% at baseline). Students indicated that the ‘Written Report Template’ was the most helpful of the changes, followed by the ‘Tips and Hints’ page. Half the students reported they received early feedback on their topic.

A few clinicians had still not seen the QIP video. Some reported delays in receiving ‘Topic Selection Forms’ from students. Most agreed students provided the required information to a satisfactory standard and that the form resulted in a more efficient process. Half indicated that their hospitals had a list of audit topics from which the students could select. Three-quarters of the clinicians agreed that the introduction of the ‘Written Report Template’ had resulted in a higher quality of reports.

Improvement in student knowledge on QI, evidenced by the quality of QIP oral presentation and the final audit report, was assessed using a pre-agreed template.

### Embedding change

The revised QIP has now been adopted at all eight clinical training sites with a coordinator employed at the university to facilitate coordination across sites, develop a learning network and identify other opportunities to improve the programme.

## Discussion

This study established the feasibility of a QI education programme within a four week O&G clinical attachment that provides an important experiential learning opportunity for Year 6 medical students without imposing an unsustainable burden on clinical services. The educational value of the QIP was highlighted with medical students and clinicians appreciating it as a means of learning QI skills in the real world. Consistent with our findings of improvements in final report quality and knowledge about QI, positive QI learning outcomes from a four-week clinical placement have been previously demonstrated [18, 19].

The major limitation of this study was that only students who had O&G attachments during the study period were included and therefore, the findings may not be representative of all students. Likewise, it is uncertain if voices of the clinical trainers and hospital personnel disengaged with the QIPs were reflected adequately. Despite these limitations, the study provides insights on the value of experiential learning of QI within the complex context of a medical education programme undergoing change. The focus groups occurring before questionnaires may have introduced some bias, although the impact is likely to be minimal as we used the two data collection methods to complement each other. The absence of student input into the process and product evaluation phase of the project from the two smaller centres is a limitation as it means that any issues unique to clinical placements in smaller centres have not been captured. In the trial of the revised QIP model, students completed the QIP in the shorter four week period, however, clinical work continued for five weeks, which meant the study could not assess the impact on clinical training demands in a shorter period.

The strength of this study is that it used Stufflebeam’s CIPP model which is consistent with the complexity theory and flexible enough to be used to support ongoing programme improvement [12]. We also used multiple stakeholder feedback on the programme and multiple data collection methods to address the pre-determined evaluation questions. Although the study period was limited, this meant that we were able to capture feedback from the same clinicians before and after implementing the revised QIP programme.

The benefits of experiential QI training as opposed to just educational sessions on QI are many. Students who actively engage in QIPs learn first-hand about logistical challenges, administrative issues and financial constraints and human factors [2, 20]. Key characteristics of successful QI curricula in medical education that have been previously identified include: attention to the interface of clinical and education systems; considered choice of QIPs for the trainee; and appropriately trained local faculty [3]. The positive response from both students and clinicians in this study is encouraging. However, it would be useful to track students once they are practising clinicians to see if they continue to integrate QI into their practice. This is particularly relevant as conducting a clinical audit is now a requirement for annual medical registration in New Zealand [21]. RANZCOG Fellows are also required to engage with the quality cycle to obtain Practice Review and Clinical Risk Management points for their Continuing Professional Development [22].

QI is an important facet of medical education ensuring the delivery of affordable, high quality and user-friendly healthcare [1, 2, 4, 13]. Providing opportunities for student and junior doctors to develop skills in QI will help ensure they are equipped to lead improvements in their future clinical practice [4, 6, 12, 13]. These skills are essential to ensure clinicians can critique the healthcare settings in which they work, [15] by becoming acquainted with evidence-based guidelines, able to critically evaluate the outcome of an audit and utilise this knowledge to improve care [16]. Clinicians in this study also perceived that the student QIPs resulted in better patient care in their women’s health services. Future research could include evaluating patient outcomes in O&G services where QIPs have been completed and where improvement suggestions have been implemented.

## Conclusion

This study established the feasibility of Year 6 medical students conducting real-life QI within the constraints of a limited O&G clinical attachment of four weeks. Students found the experiential learning of QIPs important for their future practice. Clinical trainers found the QIPs to be satisfying and saw the value in it for improving O&G service. A four week clinical rotation is adequate for medical educators to consider opportunities for including QIPs as part of student learning.

## Additional files

Additional file 1: Topic selection form for QIP. (PDF 70 kb)

Additional file 2: Template for QIP written report. (PDF 108 kb)

## Abbreviations

CIPP: Context/Input/Process/Product
O&G: Obstetrics and gynaecology
QI: Quality improvement
QIP: Quality improvement project
RANZCOG: Royal Australian and New Zealand College of Obstetrics and Gynaecology
