# Supplementary material for: Is the delivery of a quality improvement education programme in obstetrics and gynaecology for final year medical students feasible and still effective in a shortened time frame?
Source: BMC Med Educ. 2017 May 26;17:91. doi: 10.1186/s12909-017-0927-y (PMC5446706; doi:10.1186/s12909-017-0927-y)
Supplement: Supplementary file 1 — Topic selection form for QIP. (PDF 70 kb) [file 12909_2017_927_MOESM1_ESM.pdf]

Topic Selection Form for Quality Improvement Project

Part A Student details

Names ____

Hospital ______________________ Contact email __________________________________

We confirm that we will adhere to University and DHB policies around patient confidentiality and privacy. Any data saved on student hard drives or data sticks will be protected for the duration of the project and deleted at the end of the project.

____ ______________________
Signatures Date

Part B Project details

Audit topic/question ____

____

The Standard ____

Sample size ____

Variables ____

Inclusion criteria ____

Exclusion criteria ____

Part C Clinical Supervisor details

Name ____

Email ____

Please submit form to your Supervisor by first Friday of the attachment. Visit the CECIL 'O & G attachment' page to access resources to assist you to complete your project

FOR SUPERVISOR USE ONLY

- o I confirm this topic would be suitable and appropriate for the QI project
- o This topic would be suitable and appropriate with the following modifications:
  -
  -
  -

____________________________________ ______________________ _________________
Name Signature Date

Please return completed form to student by Tuesday of second week of attachment

 © University of Auckland
