# Supplementary material for: Is the delivery of a quality improvement education programme in obstetrics and gynaecology for final year medical students feasible and still effective in a shortened time frame?
Source: BMC Med Educ. 2017 May 26;17:91. doi: 10.1186/s12909-017-0927-y (PMC5446706; doi:10.1186/s12909-017-0927-y)
Supplement: Supplementary file 2 — Template for QIP written report. (PDF 108 kb) [file 12909_2017_927_MOESM2_ESM.pdf]

**Template for Quality Improvement Project Written Report**

*This report should be maximum 3 pages of A4 at 12 font (including key figures and tables), and presented in bullet point format. The aim of this report is to provide feedback to the clinical service so that they might be able to use your work for quality improvement.*

***Please*** *use the headings and include the information requested below.*

Visit the CECIL O & G attachment page to access resources to assist with your project.

| |
|---|
| **Title:** *Provide a succinct and relevant title so that an interested clinician could search and find your project, e.g. "chlamydia screening in pregnancy"* |
| **Student names:** |
| **Clinical supervisor(s):** |
| **Category and Hospital:** *(circle the relevant category/s and hospital)*<br><br>**Gynaecology:** *Oncology/ Colposcopy/ Infertility/ Urogynaecology/ General*<br>**Maternity:** *Antenatal/ Intrapartum/ Postnatal/ Neonatal*<br>**Hospital:** *Auckland/Hamilton/Middlemore/North Shore/Rotorua/Tauranga/Waitakere/Whangarei* |
| **Standard:** *In 1-2 sentences describe the standard/criterion, target, e.g. "100% of pregnant women should have chlamydia screening in pregnancy." DO NOT INCLUDE THE RANZCOG DIAGRAM* |
| **Methods:** *This section should provide enough detail for someone to replicate the project at a later date. It should include:*<br>• *description of the sample (including inclusion/exclusion criteria, sample size, sampling method)*<br>• *data source(s)*<br>• *definition of any derived data variables.* |
| **Results:** *should include:*<br>• *a table describing the sample*<br>• *data and figures that directly address the standard*<br>• *a run chart/Pareto chart/ Ishikawa diagram as appropriate to illustrate further analysis if the standard was not met.* |
| **Limitations:** *Describe briefly any limitations of your audit that are relevant to the service understanding the validity of the work you have done. For example, are the results generalisable beyond your sample? Were there a lot of missing data? Are the data contemporaneous?* |
| **Interpretation of findings:** *This section should include relevant points on what the findings mean, how they fit in the context of the service, along with one recommendation that you think the service could implement right away.* |

 © University of Auckland
